# YAP triggers the Wnt/β-catenin signalling pathway and promotes enterocyte self-renewal, regeneration and tumorigenesis after DSS-induced injury

**DOI:** 10.1038/s41419-017-0244-8

**Published:** 2018-02-02

**Authors:** Feihong Deng, Liang Peng, Zhijun Li, Gao Tan, Erbo Liang, Shengbo Chen, Xinmei Zhao, Fachao Zhi

**Affiliations:** 1 Guangdong Provincial Key Laboratory of Gastroenterology, Department of Gastroenterology, Institute of Gastroenterology of Guangdong Province, Nanfang Hospital, Southern Medical University, Guangzhou, Guangdong Province 510515 China; 2 Department of Gastroenterology, The First Affiliated Hospital of Guangzhou Medical University, Guangzhou, Guangdong Province 510120 China

## Abstract

Impaired epithelial regeneration is a crucial pathophysiological feature of ulcerative colitis (UC). Yes-associated protein (YAP1) appears to control cell proliferation and differentiation. In this study, we sought to identify the roles of YAP in intestinal epithelial cell (IEC) self-renewal, regeneration and tumorigenesis. We first observed that YAP was significantly reduced in 62.5% (45/72) of human UC tissues and it was dramatically enhanced during epithelial regeneration in a murine colitis model. Using lentiviral infection, we established a YAP-overexpression (YAP^WT^) mouse model. We then found that after tissue injury, YAP^WT^ mice had increased epithelial cell self-renewal capacity and drastically restored intestinal crypt structure. Strikingly, these mice were more susceptible to colitis-associated cancer (CAC) in chemically induced carcinoma. Mechanistically, YAP and β-catenin showed increased nuclear co-localization during regeneration after inflammation. Overexpressing YAP significantly improved IEC ‘wound-healing’ ability and increased the expression of both β-catenin and the transcriptional targets of Wnt signalling Lgr5 and cyclin D1, whereas silencing β-catenin in YAP^WT^ cells attenuated this effect. Remarkably, we observed that YAP could directly interact with β-catenin in the nucleus and formed a transcriptional YAP/β-catenin/TCF4 complex; Lgr5 and cyclin D1 were confirmed to be the target genes of this complex. In contrast, cancer cell proliferation and tumour development were suppressed by the phospho-mimetic YAP mutant. In summary, nuclear YAP-driven IEC proliferation could control epithelial regeneration after inflammation and may serve as a potential therapeutic target in UC. However, excessive YAP activation promoted CAC development.

## Introduction1

One essential function of the Intestinal epithelium is to act as a physical barrier that hampers luminal antigens such as the intestinal microbiota interacting with the internal milieu, while maintaining the absorption of nutrients and ions^1,2^. A contiguous layer of cells has a pivotal role in the epithelial barrier function. During homeostasis, intestinal epithelial cells (IECs) self-renew every 3–5 days^3^. Upon surface damage, an intricate repair process is triggered to restore the structural and functional integrity of the impaired tissue within several minutes. This process is also called ‘epithelial restitution’, is particularly important in the resealing of the damaged epithelium^4^, following by intestinal stem cells (ISCs) activation, proliferation and differentiation^5^.

Ulcerative colitis (UC) is a subcategory of inflammatory bowel disease (IBD) with high-risk of colitis-associated cancer (CAC)^6^. Two key pathophysiological features of this disease are the dysregulation of innate immune system and impaired epithelial regeneration^7^. Although, immunosuppressive therapies in treatment of UC are advancing, the poor clinical consequences and variable complications still remain UC as a challenge to physicians. For both pathophysiological features, mucosal healing has recently been regarded as a key treatment goal to UC^8^.

Multiple pathways including Wnt/β-catenin, Notch, Hippo/Yes-associated protein and transforming growth factor-β (TGF-β) are involved in the regenerative epithelial process^9,10^. The Hippo pathway is particularly essential in control of organ size and tissue growth^11,12^. YAP (YAP1 or YAP65) is a core component of the Hippo pathway and a candidate oncogene in humans^13^. When Hippo is active, the LATS1/2 kinases of the Hippo pathway phosphorylate YAP, leading to YAP cytoplasmic retention and degradation by β-TrCP ubiquitin ligase^11,14^. Upon nuclear translocation, YAP binds to transcription factors (mainly TEAD family proteins) and acts as a transcriptional co-activator to initiate the transcription of target genes^4,15^. YAP regulates EGFR-dependent regenerative program via elevated Areg (acts as a YAP/TEAD target gene) after exposure to irradiation^16^. Meanwhile, biological activation of the Notch pathway, which is important in epithelial cell proliferation/differentiation^17^, was alternative driven via YAP interaction with Notch ligands^18^.

In addition to YAP in epithelial cells, the Wnt signalling pathway is a critical regulator of stem cell maintenance, cell fate and proliferation^19,20^. When inactive, cytosolic β-catenin is recruited into the adenomatous polyposis coli (APC)/Axin/GSK3/CK1 destruction complex, resulting in its phosphorylation by GSK3 and CK1 and degradation by the β-Trcp ubiquitin ligase. When activated, β-catenin escapes the regulation of the destruction complex and triggers transcription by subsequent nuclear accumulation and interaction with TCF/LEF transcription factors^21,22^. Establishing the ISC compartment requires Wnt signalling that deletion of TCF4 expression caused foetal mice to lack proliferative crypts^23^. Moreover, the conditional knockout of β-catenin or TCF4 results in crypt proliferation deficiency in adult mice, which suggested that maintenance of IECs proliferation was Wnt-dependent^24^^,^^25^.

The overlap between YAP and Wnt regulation in IEC proliferation imply that these factors do not work independently and might have a potential link, but the available mechanism has not been investigated yet. In this study, we demonstrate that YAP triggers Wnt/β-catenin signalling, which stimulates epithelial cell proliferation and not only facilitates enterocyte self-renewal and crypt regeneration after colitis, but also promotes CAC development through chronic inflammation and excessive regeneration.

## Materials and methods

### Human colon samples

Two groups of human specimens were procured with approval from the Institute Research Medical Ethics Committee of Nanfang Hospital. One group comprised paraffin-embedded sections of biopsy specimens (*n *= 72 UC and *n* = 21 normal tissues) from the Department of Pathology at Nanfang Hospital. These sections were used for analysing immunohistochemical (IHC) expression. The other matched specimens (*n *= 15) were routine colonoscopy biopsies procured from the Department of Gastroenterology at Nanfang Hospital. These fresh specimens were used for Western blotting and RT-PCR analysis. The corresponding clinical data were recorded from the medical records. Patient characteristics and histological data are shown in Table 1.

**Table 1:** Patient characteristics

Characteristic	Normal control (*n *= 21)	Inactive UC (*n *= 30)	Active UC (*n *= 42)
Gender			
Male	13	19	22
Female	8	11	20
Age (years ± SD)	44.3 ± 12.8	44.7 ± 19.2	47.9 ± 12.2
Disease duration (years ± SD)	_	3.2 ± 5.3	4.2 ± 4.8
Extent of disease			
Extensive colitis	_	10	17
Left-sided colitis	_	12	12
Proctitis	_	8	13
Treatment			
TNF antagonist use	_	5	7
Aminosalicylates	_	20	28
Immune modulator use	_	4	8
Corticosteroids	_	11	20
None	_	3	3
Pathological severity			
Grade 0–1	_	30	_
Grade 2–5	_	_	42

### Reagents and antibodies

Recombinant Wnt-3a and DKK1 were purchased from R&D Systems (Abingdon, UK); AOM (Azoxymethane), fluorescein isothiocyanate (FITC)-dextran (40,000 kDa) and 5-bromo-2′-deoxyuridine (Brdu) were from Sigma (St. Louis, MO); and dextran sodium sulfate (DSS; molecular weight (MW) 40,000–50,000 kDa) was from MP Biomedicals (Santa Ana, CA). The following antibodies were purchased: YAP, phosphor-YAP S127, β-catenin, β-Trcp, Brdu and Ki67 (Cell Signaling Technology, Boston, MA); cyclin D1, LATS2, DVL2 Caspase-3 and PCNA (Proteintech, Rosemont, USA); Lgr5 and TCF4 (Abcam, Cambridge, MA); and c-myc, CK20, glyceraldehyde 3-phosphate dehydrogenase (GAPDH) and histone H3 (Santa Cruz, Dallas, Texas).

### Plasmids, siRNA and transfection

The lentiviral expression vectors overexpressing wild-type YAP protein and the YAP mutant were purchased from Genechem (Shanghai, China). The vectors were GV358, which contained the component order Ubi-MCS-3FLAG-SV40-EGFP-IRES-puromycin. The primer sequences for YAP1 were as follows: Human YAP1 forward: 5′-GAGGATCCCC

GGGTACCGGTCGCCACCATGGATCCCGGGCAGCAGCCGC-3′ and reverse: 5′-TCCTTGTAGTCCATACCTAACCATGTAAGAAAGCTTTC-3′. Mouse YAP1 forward: 5′-GAGGATCCCCGGGT ACCGGTCGCCACCATGGAGCCCGCGCAACAG-3′ and reverse: 5′-TCCTTGTAGTCC

ATACCTAACCACGTGAGAAAGCT-3′. Both YAP fragments harbouring the S127D (serine to alanine at residue 127) and the S112D (serine to alanine at residue 112, corresponding to S127 of human YAP) mutations were inserted into the vector GV358 to construct the recombinant lentivirus plasmid. Stable YAP-WT-, YAP-mutant- and empty control vector-transfected clones were selected using medium containing puromycin (2 μg/ml) for 2–3 weeks. The pcDNA3.1-β-catenin-myc plasmid was kindly provided by Dr. Gengtai Ye (Southern Medical University, Guangzhou, China). Specific YAP and β-catenin small interfering RNAs (siRNAs) and control siRNAs were obtained from Genepharma (Shanghai, China). The sequences of siRNAs were detailed in Supplementary Table 2. Transfection with plasmids or siRNAs was completed using Lipofectamine 3000 (Invitrogen, Carlsbad, CA) as described by the manufacturer. Infection of the above cells was confirmed by immunoblot analysis.

### Cell culture

The FHC, DLD1, HT29 and HEK293T cell lines were obtained from the American Type Culture Collection (ATCC, Manassas, VA). Cell lines were cultured in Dulbecco’s modified Eagle’s medium (DMEM) supplemented with 10% fetal bovine serum (FBS) at 37 °C with 5% CO_2_. For DSS-induced inflammation in vitro, FHC cells were treated with 1% DSS for 0 or 4 h after an overnight starvation period in serum-free medium, followed by 1 h (4 + 1 h), 2 h (4 + 2 h) or 4 h (4 + 4 h) of incubation in reduced serum culture medium; Wnt activation was accomplished by treating FHC cells with Wnt3a (100 ng/ml) for the indicated time.

### Nuclear and cytosol fractionation

Nuclear and cytosol fractions were obtained using a Nuclear and Cytoplasmic Protein Extraction Kit (Beyotime, China) according to the manufacturer’s instructions. Briefly, 1 × 10^7^ cells were washed with phosphate-buffered saline (PBS), collected in 200 µl of cytoplasmic protein extraction agent A/protease inhibitor buffer and then left on ice for 10–15 min. Cells were then incubated in 10 µl of cytoplasmic protein extraction agent B, followed by centrifugation at 12,000 *g* for 10 min at 4 °C to pellet nuclei away from the cytoplasm. The nuclei were resuspended in 50 µl of nuclear protein extraction buffer and agitated for 30 min on ice. After centrifugation at 12 000 *g* for 10 min at 4 °C, the supernatant was collected as the nuclear extract for subsequent analysis.

### Co-immunoprecipitation and western blot analysis

Equal amounts of protein samples were loaded onto 10 to 12% SDS-polyacrylamide gel electrophoresis gels and transferred onto polyvinylidene difluoride membranes (Bio-Rad). Protein blots were incubated with the indicated primary antibody and then with the appropriate secondary antibody, followed by detection with the enhanced chemiluminescence detection system.

For immunoprecipitation, cells were lysed in buffer containing 50 mM Tris-HCl (pH 7.5), 150 mM NaCl, 5 mM EDTA, 1% Triton X-100, 1 mM phenylmethylsulfonyl fluoride and cocktail protease inhibitors followed by centrifugation at 12,000 *g* for 30 min at 4 °C. Lysates (200 μg) were then precipitated with 5 µg of the indicated antibody or control IgG plus 20 µl of protein A/G agarose (Santa Cruz) for 2 h. After extensive shaking, immune complexes were then washed three times in lysis buffer and subjected to western blot analysis using the indicated antibodies.

### Chromatin immunoprecipitation assays

Quantitative chromatin immunoprecipitation was performed with a Pierce Agarose ChIP Kit (Thermo Fisher Scientific) according to the protocol, FHC-EV, FHC-YAP^WT^ and FHC-YAP^S127D^ cells were crosslinked for 10 min using a final concentration of 1% formaldehyde in the media. After sonication, the chromatin solution was diluted in chromatin immunoprecipiattion (ChIP) lysate and incubated with either anti-TCF4 antibody or normal IgG. ChIP Grade Protein A/G plus Agarose (20 μl) was added to each fraction and rotated at 4 °C. Then the antibody/protein/DNA complex was washed using elution buffer. The DNA–protein crosslinks were reversed by heating at 65 °C for 4 h. DNA was finally purified and subjected to real-time PCR. The sequences of the PCR primers used were as follows: Lgr5 forward 5′-GGAGGGGACAAGTGGAGGG-3′ and reverse 5′-CAGTGGCGGTGCGCC-3′, and cyclin D1 forward 5′-AAGAGTCTCCAGGCTAGAAGGACA-3′ and reverse 5′-AGTTAAAGGGATTTCAGCTTAGCATG-3′.

### Confocal microscopy

FHC cells were plated at sterile coverslips in culture dishes. Twelve hours later, cultures were subjected to overnight starvation with serum-free medium prior to treatment with 1% DSS for 4 h. Cells were washed thrice with PBS, followed by fixation with 4% paraformaldehyde for 15 min at room temperature. The fixed cells were washed three times and permeabilized with 0.5% Triton X-100 in PBS at room temperature for 10 minutes. Cells were then left in 3% bovine serum albumin/PBS blocking buffer for 30 min, followed by an incubation with primary antibody overnight at 4 °C. Then with three times washing, cells were incubated with secondary fluorescent antibodies for 1 h and counterstained with DAPI (4',6-diamidino-2-phenylindole). Cells were visualized with an FV1000 confocal laser scanning microscope (Olympus, Japan). Images were analysed with FV10-ASW 3.0 Viewer software.

### Quantitative real-time PCR

Total RNA was extracted from cells and tissues using TRIzol (Takara, Japan), and cDNA synthesis was performed with a reverse transcriptase kit (Takara) according to the manufacturer’s protocol. Then, SYBR Green Premix Ex Taq was used for subsequent quantitative real-time PCR amplification on an LC480 system. GAPDH was used as the internal control. Gene-specific primer sequences are listed in Supplementary Table 1.

### Histochemistry and immunohistochemistry

Mouse colon samples were fixed in 4% paraformaldehyde for 24 h and embedded in paraffin; 5 μm-thick sections were used for haematoxylin and eosin or IHC staining. For IHC, deparaffinized sections were performed quenching of endogenous peroxidase activity, antigen retrieval and subsequent blocking procedures. Slices were incubated in the primary antibodies of interest at 4 °C overnight, followed by incubation with biotinylated secondary antibody for 2 h at room temperature. The immunosignal was visualized via a DAB kit. The histological inflammation score and tumour classification were independently analysed by two senior pathologists.

### Cell proliferation, wound healing and colony-formation assays

Cell proliferation was measured using a Cell Counting Kit-8 (Dojindo, Tokyo) according to the manufacturer’s instruction. Briefly, 100 µl of cells were plated at a concentration of 2000 cells/well in 96-well plates. CCK-8 working buffer (10 μl) was added into the cultures and incubated at 37 °C for 1 h. Colorimetric measurements were performed at 450 nm on a microplate reader.

For scratch assays, cell migratory activity was visualized after transfection with targeted plasmids and (or) siRNAs. The cell monolayer was scratched with a 10 μl pipette tip. Cells that migrated towards the scratches were imaged at the indicated times.

To study colony formation, 500 cells were seeded in 6-well plates. After 7-day incubation, colonies were obtained, fixed with 4% paraformaldehyde and then stained with 0.05% crystal violet; the number of colonies was calculated, followed by extensive washing in PBS.

### Cell apoptosis

Assessment of APC Annexin V-labelled cell apoptosis was measured according to the manufacturer’s protocol (KeyGEN BioTECH, Nangjing, China). FHC-EV and FHC^WT^ Cells were seeded on six-well plates and treated with 1% DSS for 12 h, then cells were suspended in 500 μl binding buffer containing 5 μl APC Annexin-V and 5 μl Propidium Iodide (PI). Cells were analysed by a fluorescence-activated cell sorting (BD Biosciences). A total of 10,000 cells were analysed per determination. Cells were considered apoptotic when they manifested early apoptosis (Annexin-V positive, PI negative) or late apoptosis (Annexin-V positive, PI positive).

### Mouse work

BALB/c mice were purchased from the Medical Experimental Animal Centre of Southern Medical University. All experiments were performed with the approval of the Animal Experimentation Committee of the Southern Medical University (2016023) and in accordance with institutional regulations. For the YAP genetically manipulated model, 6- to 8-week-old male mice were infected with mouse YAP^WT^ or YAP^S112D^ lentivirus (1 × 10^7^ units per mouse) in a total volume of 100 µl by intraperitoneal injections, the empty vector (EV) was used as the internal control. Three days later, gene expression in mice was first analysed to insure the successful infection and then different model constructions were subsequently performed. For chronic model, mice were treated with lentivirus intraperitoneally twice a week.

For DSS-induced colitis and regeneration, mice underwent intestinal inflammation by receiving water with 3% DSS (MW 40,000–50,000, MP Biomedicals) for 5 consecutive days (5d), and followed by 5 days normal drinking water (5 + 5d), which was defined as regeneration after colitis. In this model, body weight and disease activity index (DAI) of mice were monitored every day. Mice were killed at the time points indicated in the text.

To generate chemically induced colorectal carcinoma, mice were intraperitoneally injected with 10 mg/kg body weight AOM (Sigma). One week later, mice were treated with 3% DSS in distilled water for 7 days, followed by 14 days of normal drinking water as previously described^26^. This cycle was repeated three times. Mice were killed at the time points indicated in the text.

### Isolation of primary colonic epithelial cells from mice

After the mice were killed according to the previously related protocol, the colons were obtained, cut the colon open lengthwise and rinsed with ice-cold PBS. Crypts were obtained after incubating intestines at 37 °C in 5 mM EDTA for 20 min with extensive shaking at 200 r.p.m. FBS/DMEM (10%) was added to neutralize the digestion, then passed buffer/crypt solution through a 70 μm cell strainer and collected in a 50 ml centrifuge tube. The pellet was collected by centrifuging the filtered crypts at 1,000 *g* for 10 min at 4 °C.

### Histological analysis

Human UC severity was graded from 0 to 5 according to Geboes criteria^27^. Immunoreactivity was assessed from immunoreactive scores (IRSs). In addition, the histological scores of mice were assessed in a blinded manner according to the previously described criteria^28^. Each quantitative measurement was evaluated from 3–5 fields of view in each independent specimen.

### Brdu-labelling assays

Mice received intraperitoneal injections of 50 mg/kg Brdu before killing; then, mouse colons were collected for paraffin sections and anti-Brdu IHC staining. The number and location of Brdu-positive cells were assessed in the different crypt samples.

### Intestinal permeability assay

Intestinal permeability was assessed through the oral administration of FITC-labelled dextran as previously described^29^.

### Statistics

Data are expressed as the means ± SEMs. Significance was determined using Student’s t-test, one-way or two-way analysis of variance, as appropriate, for multiple comparisons by Prism 5 (GraphPad Software, USA). Statistical significance was defined as a *P-*value < 0.05.

## Results

### YAP expression was decreased in human UC tissues

**Fig. 1:**
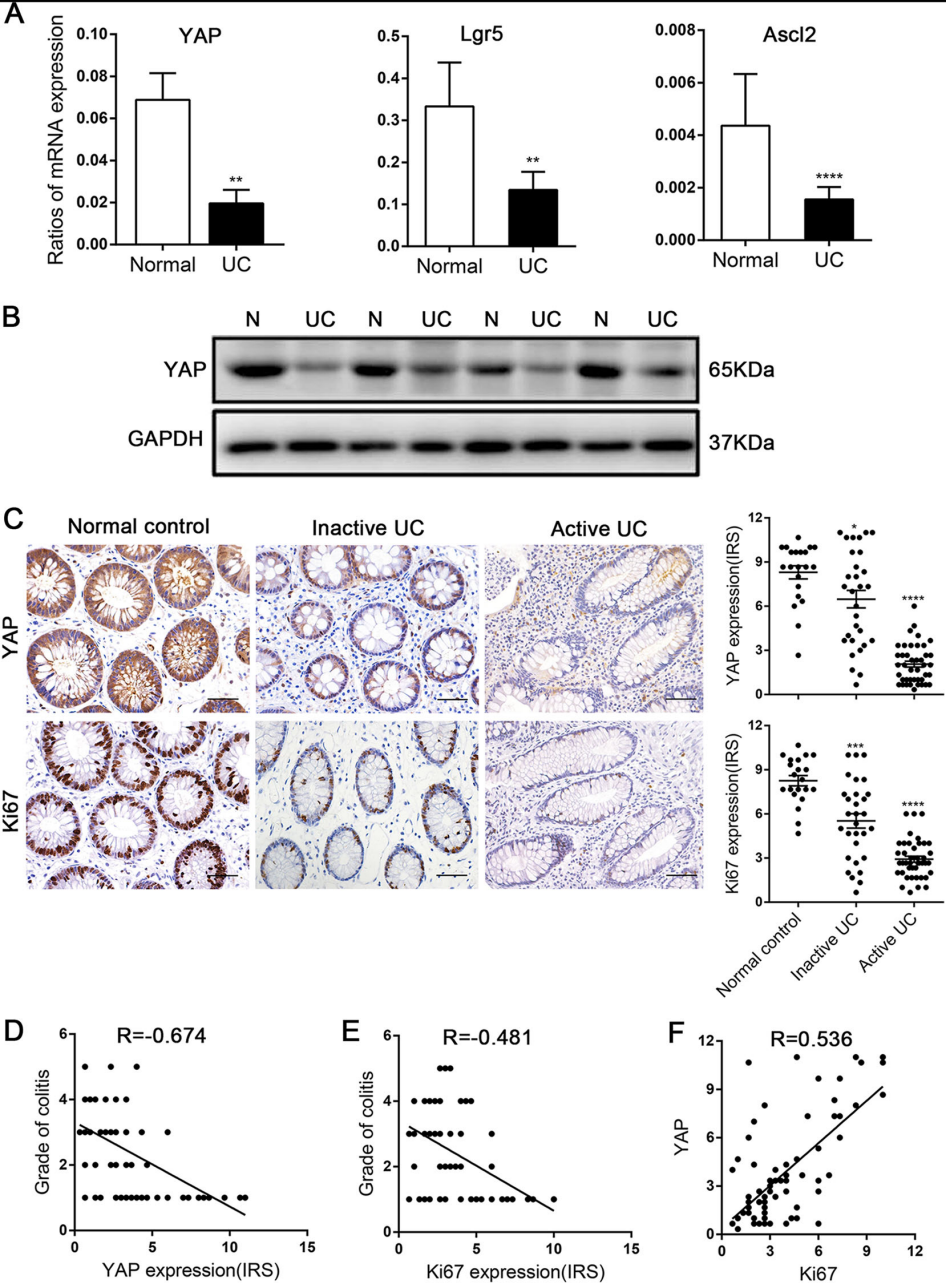
YAP expression was down-regulated in human ulcerative colitis (UC). (**a**) mRNA expression levels in ulcerative colitis and matched normal tissues (*n* = 15), as determined by real-time PCR. GAPDH mRNA expression was used as the internal control. Data represent the means ± SEMs. **P < 0.01, ****P < 0.0001. (**b**) Western blot analysis of total YAP protein expression level in UC and corresponding normal tissues (*n* = 15). N, normal colon; UC, ulcerative colitis. (**c**) Immunohistochemical staining of total YAP in normal controls (*n *= 21), inactive UC (*n* = 30) and active UC (*n* = 42) tissues (× 400). Data for the scatter diagrams were generated from immunoreactive scores (IRSs). Scale bars = 20 μm. Data represent the means ± SEMs. *P < 0.05, ***P < 0.001, ****P < 0.0001. (**d**) The Spearmen’s correlation between YAP expression and colitis severity (*r* = – 0.674, *p* < 0.0001). (**e**) The Spearman’s correlation between Ki67 expression and colitis severity (*r* = – 0.481, *p* < 0.0001). (**f**) The Spearman’s correlation between YAP and Ki67 expression in epithelial cells of patients with UC (*r* = 0.536, *P* < 0.0001)

We first examined the expression of proliferation-related genes in fresh specimens obtained from colonoscopies of UC tissues and found that the YAP mRNA level was decreased in 80% (12/15) of paired UC tissues, concomitant with declined ISC signature genes, *Lgr5* and *ascl2* (Fig. 1a). Meanwhile, total YAP protein expression was also lower in UC tissues than in the matched normal tissues (Fig. 1b). IHC analysis of YAP localization in human normal colons showed that it accumulated in both the nucleus and cytoplasm of epithelial cells. YAP was significantly reduced in 62.5% (45/72) of UC tissues, especially in active UC, and was undetectable in 18.1% (13/72) of these specimens. In parallel, Ki67 expression in patients with active UC showed a significant decrease compared with that in patients with inactive UC or in control patients (*p* < 0.0001, Fig. 1c). In addition, Spearman’s correlation analyses revealed that both YAP and Ki67 expression in epithelial cells correlated negatively with colitis severity (*R* = – 0.674, *p* < 0.0001; *R* = – 0.481, *p* < 0.0001, Fig. 1d,e) and YAP accumulation was positively associated with Ki67 expression in UC tissues (*R* = 0.536, *p* < 0.0001, Fig. 1f). Patients in long-term remission with no endoscopic signs of colitis were defined as inactive UC (Geboes histology score 0 or 1). Patients in the acute stage of the disease with endoscopically visible signs of colitis were defined as active UC (Geboes histology score ≥ 2)^30^. These data demonstrated that epithelial cell proliferation was obstructed in the development of human UC and could be mediated by YAP.

### YAP expression was upregulated during epithelium regeneration in murine colitis model

**Fig. 2:**
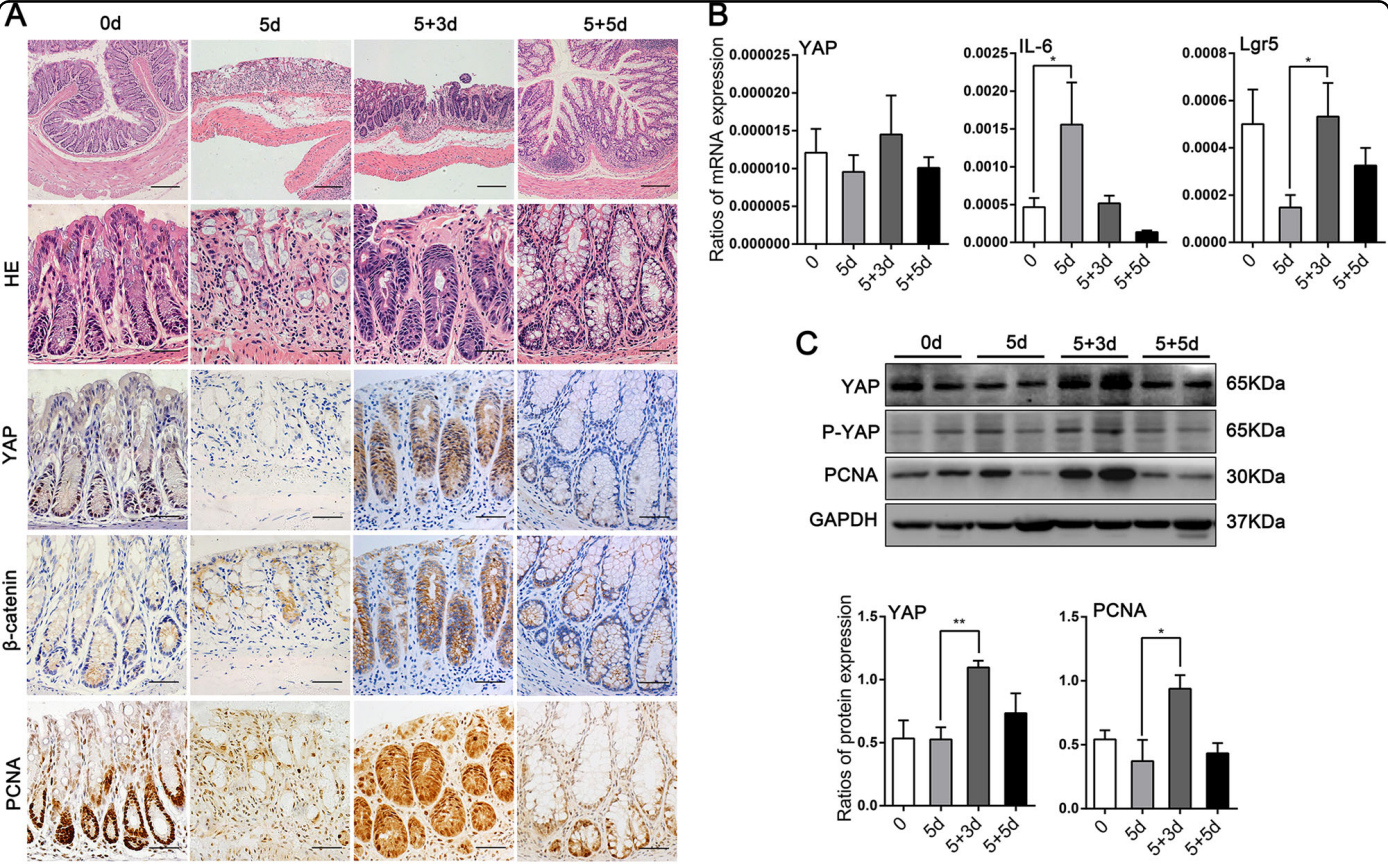
YAP expression was enhanced during epithelial regeneration following DSS-induced colitis in mice. (**a**) H&E (upper × 100, bottom × 400) and immunohistochemical staining (× 400) in colons of BALB/c mice at day 0 and 5 days after treatment with 3% DSS, followed by 3 days (5 + 3d) or 5 days (5 + 5d) of normal drinking water (*n* = 4 each). Scale bars = 200 μm. (**b**) mRNA expression levels were detected at the indicated times by real-time PCR. Data represent the means ± SEMs. *P < 0.05. (**c**) Immunoblot (IB) analysis of the indicated proteins in primary colon cells prepared from mice killed at the indicated times, followed by densitometric analysis of the blots. *n* = 4. Data represent the means ± SEMs. *P < 0.05, **P < 0.01

As previously reported, YAP is particularly important in hepatocyte regeneration after liver injury^31^. To examine the role of YAP during intestinal homeostasis, inflammation and regeneration, we took advantage of the well-established DSS-induced colitis and repair model. During homeostasis, active IEC proliferation was displayed at the crypt base. Five days of 3% DSS exposure led to the destruction of most colonic crypts with severe inflammatory cells infiltration. However, after 3 days of DSS withdrawal, crypts consisted of hyperproliferative epithelial cells that extended the entire crypt length and the ulcer area was clearly reduced. Crypt structure and the distribution of differentiated cells were largely restored 5 days after the removal of DSS (Fig. 2a).

In this murine model, we examined the temporal and spatial modulation of YAP in colonic mucosa. During homeostasis, YAP was specifically present in the colonic crypt base. After DSS treatment, the expression of YAP was dramatically diminished in crypts, with certain cells displaying weak cytoplasmic localization. After DSS withdraw for 3 days, a tremendous restoration of YAP expression was detected, which extended throughout the whole crypt with both cytoplasmic and nuclear accumulation (Fig. 2a), and proliferation-related markers were up-regulated (Fig. 2b,c). This upregulation in YAP protein expression might be due to post-transcriptional control since there was not a significant increase in YAP mRNA expression during regeneration (Fig. 2b). By 5 days, YAP expression was detected in the crypt base again, at almost identical levels as those in normal colon tissue (Fig. 2a). Therefore, the real-time loss and regain of YAP in crypt cells upon DSS-induced colitis suggested its particular role in IECs proliferation, especially in the course of epithelial regeneration.

### YAP accelerated self-renewal, protected enterocytes from DSS-induced colitis and promoted epithelial regeneration after mucosal injury

**Fig. 3:**
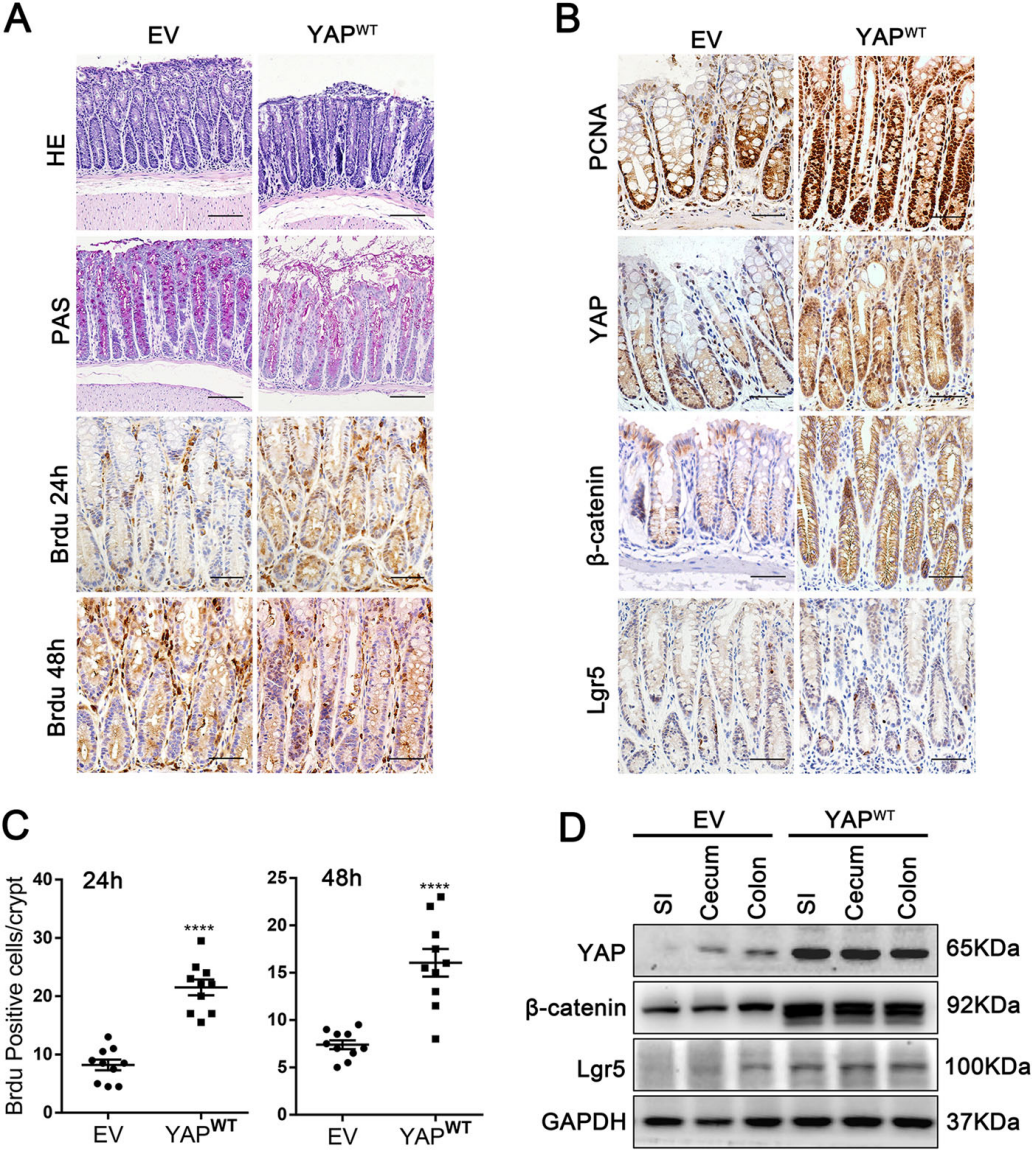
YAP overexpression increased the IEC self-renewal of mouse colonic crypts. (**a**) Paraffin-embedded colon sections from mice infected with the YAP^WT^ or empty vector (EV) lentivirus (1 × 10^7^ units per mouse) were analysed by H&E (*n* = 10 each, × 200), PAS (× 200) and Brdu staining at 24 and 48 h (× 400). Scale bars = 200 μm. (**b**) Immunohistochemical staining of PCNA, YAP, β-catenin and Lgr5 in YAP^WT^- and EV-infected mouse colons (*n* = 5 each, × 400). (**c**) Brdu-positive cells were counted in each colonic crypt after 24 and 48 h of infection. Data represent the means ± SEMs. ****P < 0.0001. (**d**) Western blot analysis of the indicated proteins in mucosal epithelium prepared from YAP^WT^ and EV mice (*n* = 4 each). SI, small intestine

To specifically validate YAP function, based on the markedly increased YAP expression in restored crypts during intestinal regeneration, we constructed a YAP overexpression model through intraperitoneal injections of YAP-wild-type lentivirus (1 × 10^7^ units once) in adult mice. We observed the increased YAP expression of mucosal epithelium including small intestine, caecum and colon 14 days after infection (Fig. 3b,d). In homeostasis, YAP overexpression caused a notable decrease in cells of the secretory lineage^4^, but no significant difference of crypt lengths (Fig. 3a). Notably, we did not observe the death of mice after infection with YAP^WT^ or EV lentirvirus for 24 weeks. A time-course study of Brdu labelling (24 and 48 h) showed not only an increase in the number of proliferating crypt cells (*p* < 0.0001) but also the activation of cell migration along the crypt axis in the YAP^WT^ mice (Fig. 3a,c). Moreover, compared with mice injected with the EV, YAP^WT^ mice also had an increased expression of proliferation-related proteins β-catenin and Lgr5 (Fig. 3b,d).

**Fig. 4:**
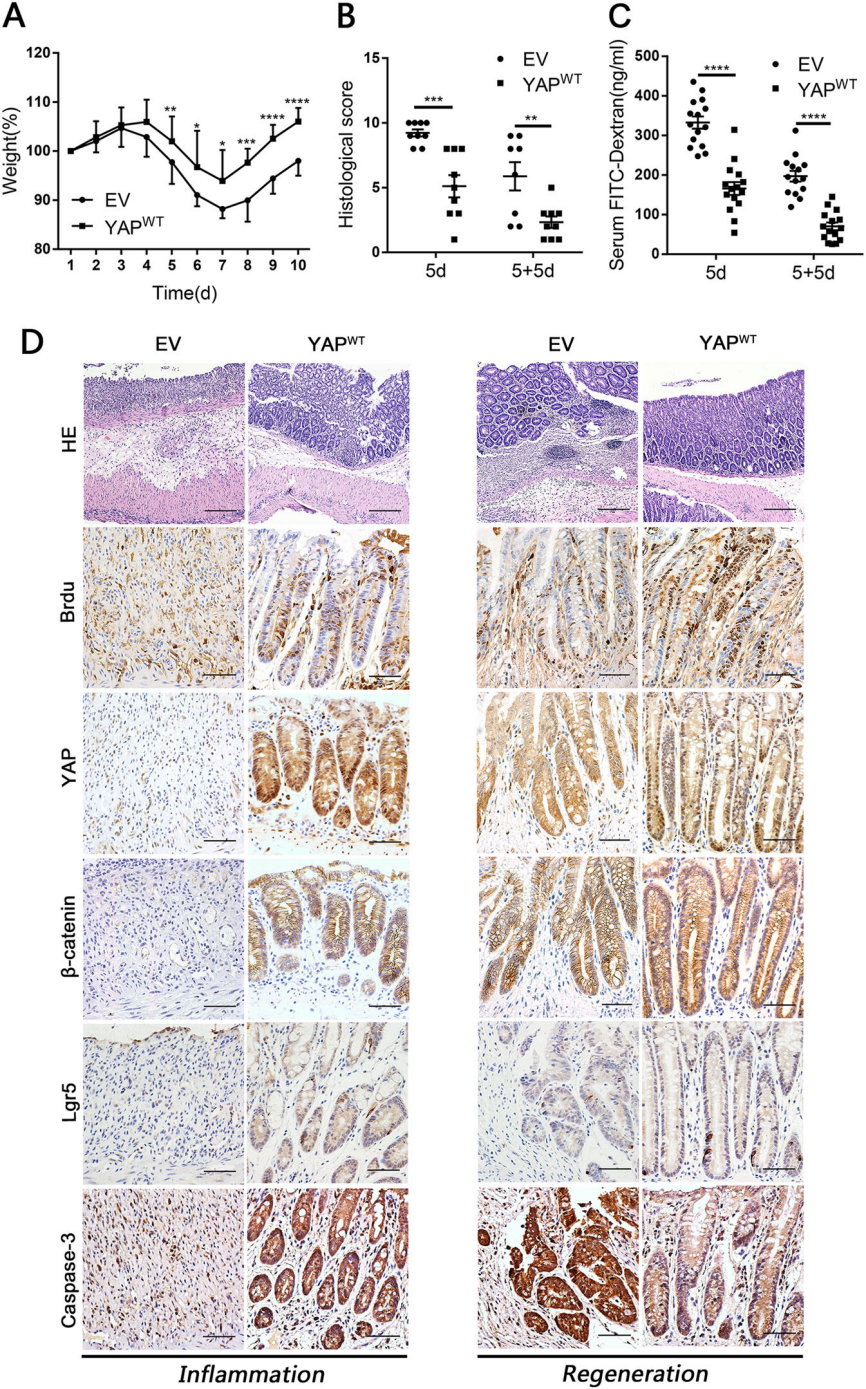
YAP overexpression protected epithelial cells against DSS-induced colitis and promoted intestinal regeneration in mice. (**a**) Body weight curves of YAP^WT^ and EV mice during the successive 5-d DSS treatment, followed by 5 days of normal drinking water. Mice were killed on 5d (*n* = 9 each) and 5 + 5d (EV *n* = 8, YAP^WT^ *n* = 9). Data represent the means ± SDs. (**b**) Histological scores in YAP^WT^ and EV mice on 5d (*n* = 9 each) and 5 + 5d (EV *n* = 8, YAP^WT^ *n* = 9). Data represent the means ± SEM. **P < 0.01, ***P < 0.001. (**c**) Serum FITC-dextran in YAP^WT^ and EV mice on 5d (*n* = 15 each) and 5 + 5d (EV *n* = 14, YAP^WT^ *n* = 15). Data represent the means ± SEM. ****P < 0.0001. (**d**) Paraffin-embedded sections of YAP^WT^ and EV mice colons were analysed by H&E (× 100) and immunohistochemical staining (× 400) at 5d (Left: inflammation) and 5 + 5d (Right: regeneration). Scale bars = 200 μm

Next, we assessed the susceptibility of YAP^WT^ mice to acute DSS-induced colitis. The overall DAI was significantly lower in YAP^WT^ mice than in EV mice, which presented with bloody stools (Supplementary Figure 1B). On average, YAP^WT^ mice had a colon length 9-mm longer (Supplementary Figure 1A) and a higher body weight than mice injected with EV (Fig. 4a). Histological assessments demonstrated that YAP^WT^ mouse crypts exhibited better structural integrity; in contrast, the control littermates presented with catastrophic crypt loss and widespread ulcers (Fig. 4b,d).

More importantly, YAP^WT^ mice rapidly regained body weight after the removal of DSS, while mice injected with the EV showed a delayed recovery (Fig. 4a). In addition, 5 days after DSS withdrawal, the structure of crypts and the gut barrier function in YAP^WT^ mice were almost restored (Fig. 4c). Moreover, colonic crypts of YAP^WT^ mice contained more Brdu and PCNA-positive proliferating cells and fewer apoptotic cells than those in EV mice (Fig. 4d and Supplementary Figure 1C and D). Collectively, these observations suggested that YAP was an essential regulator of epithelial cell proliferation that maintained normal intestinal homeostasis and mediated protection against colitis. Notably, after chemical exposure, overexpressing YAP drastically accelerated epithelial regeneration to restore the intestinal mucosal barrier in the murine model.

### YAP triggered Wnt/β-catenin signalling during epithelial cell inflammation and regeneration

**Fig. 5:**
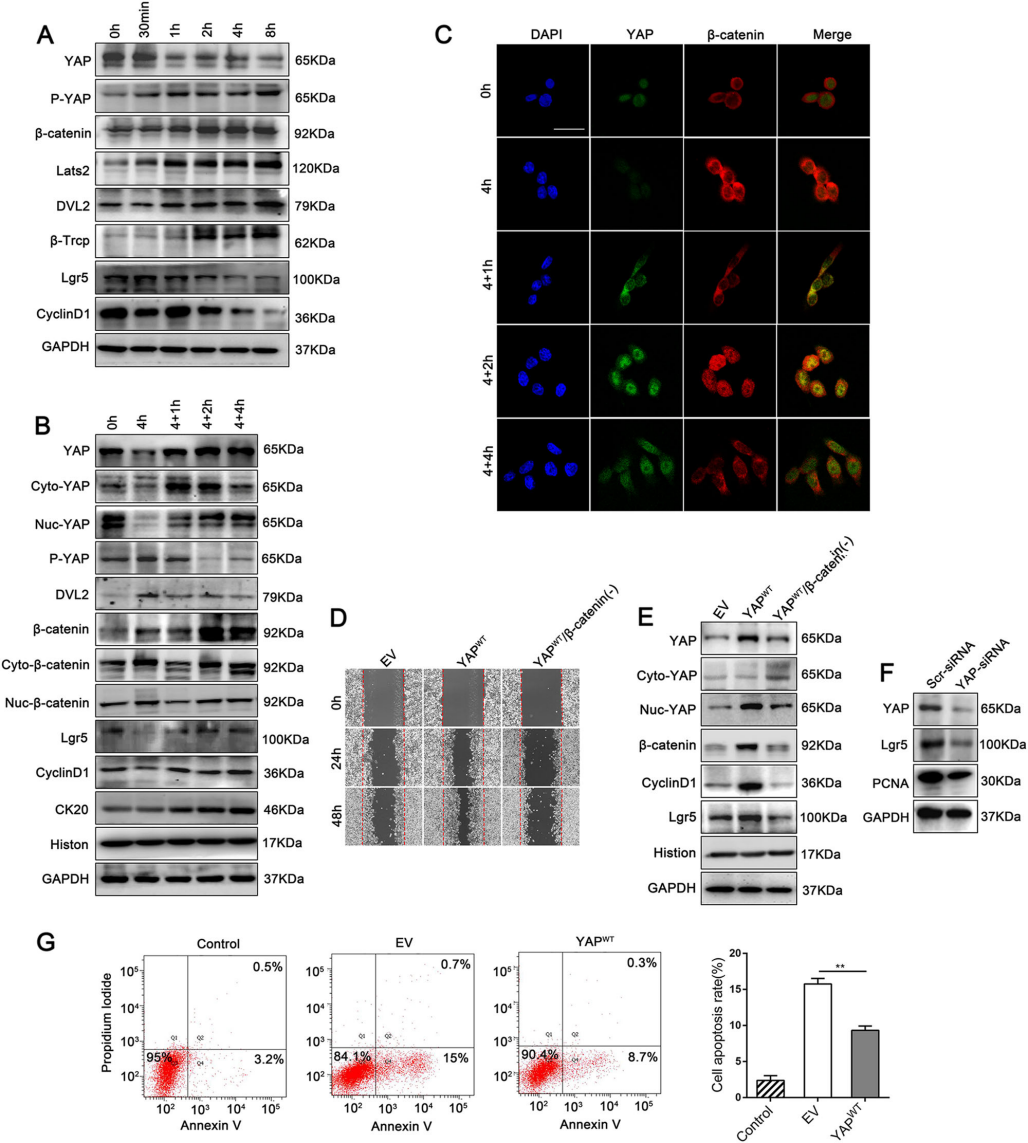
YAP expression was significantly enhanced during DSS-induced regeneration and triggered Wnt/β-catenin signalling in cell proliferation in vitro. (**a**) IB analysis of total YAP, P^S127^-YAP and Wnt/β-catenin signalling associated proteins in FHC cells. Cells were serum-starved overnight and then stimulated with 1% DSS for the indicated time. (**b**) IB analysis of total YAP, P^S127^-YAP and Wnt/β-catenin signalling associated proteins in FHC cell lines. Cells were serum-starved overnight and then stimulated with 1% DSS for 0 or 4 h, followed by 1 h (4 + 1 h), 2 h (4 + 2 h) or 4 h (4 + 4 h) of incubation in reduced serum culture medium. Cyto, cytoplasm; Nuc, nucleus. from three independent experiments. (**c**) Immunofluorescence staining for YAP and β-catenin in FHC cell lines, as determined by confocal laser scanning microscopy. Scale bars = 50 μm. (**d**) Detection of wound healing ability in YAP^WT^ and β-catenin-silenced with YAP^WT^ expression (YAP^WT^ /β-catenin( – )) in FHC cells at 0, 24 and 48 h. (**e**) IB analysis of Wnt/β-catenin related proteins expression in YAP^WT^ and YAP^WT^/β-catenin( – ) FHC cells. (**f**) IB analysis of Lgr5 and PCNA expression levels in Scr-siRNA- and YAP-siRNA-transfected HT29 cells. (**g**) Representative dot blots of APC Annexin-V versus PI. Flow cytometry analysis of apoptosis in FHC-EV and FHC-YAP^WT^ cells after incubation with 1% DSS for 12 h

As the Wnt/β-catenin signalling pathway has a particularly important role in hepatobiliary repair after chemically-induced hepatocyte injury^32,33^, to examine the relationship between YAP and β-catenin in intestinal inflammation and regeneration, we used DSS to induce epithelial cell injury in vitro, which has been done previously^34^. A time course of 1% DSS treatment in normal colonic FHC cells resulted in a progressive increase in the expression of LATS2 kinase, YAP S127 phosphorylation and E3 ubiquitin ligase β-Trcp. In contrast, YAP expression was significantly reduced, which suggested that the Hippo pathway was activated after DSS administration, leading to YAP protein degradation by the ubiquitin proteasome pathway (Fig. 5a). Strikingly, DSS triggered the activation of Wnt signalling by inducing Dvl2 and β-catenin stimulation (similar to *Wnt3a* stimulation in cells, Supplementary Figure 2A) but had no effect on Lgr5 and cyclin D1 accumulation (Fig. 5a). These data indicated that increasing the protein expression level of β-catenin alone was insufficient to initiate the transcription of Wnt signalling genes, and the loss of YAP expression caused by Hippo activation might participate in this process.

During the regeneration process, an abundant expression of cytoplasmic YAP rapidly occurred after termination of DSS administration and was gradually translocated into the nucleus; by 2 h post DSS cessation, YAP expression reached the highest amount of nuclear accumulation, and by 4 h it became a weak immune-signal again. Meanwhile, β-catenin showed clear nuclear localization, which was accompanied by up-regulated total protein expression levels of β-catenin, Lgr5 and cyclin D1 by 2 h DSS withdrawn (Fig. 5b,c), and mRNA levels of the ISC makers *Lgr5* and *Olfm4* were also clearly increased (Supplementary Figure 2B) in this period. These results suggested that in the presence of nuclear YAP, Wnt/β-catenin signalling that including expression and transcriptional function of β-catenin were concurrently triggered during epithelium cell regeneration.

**Fig. 6:**
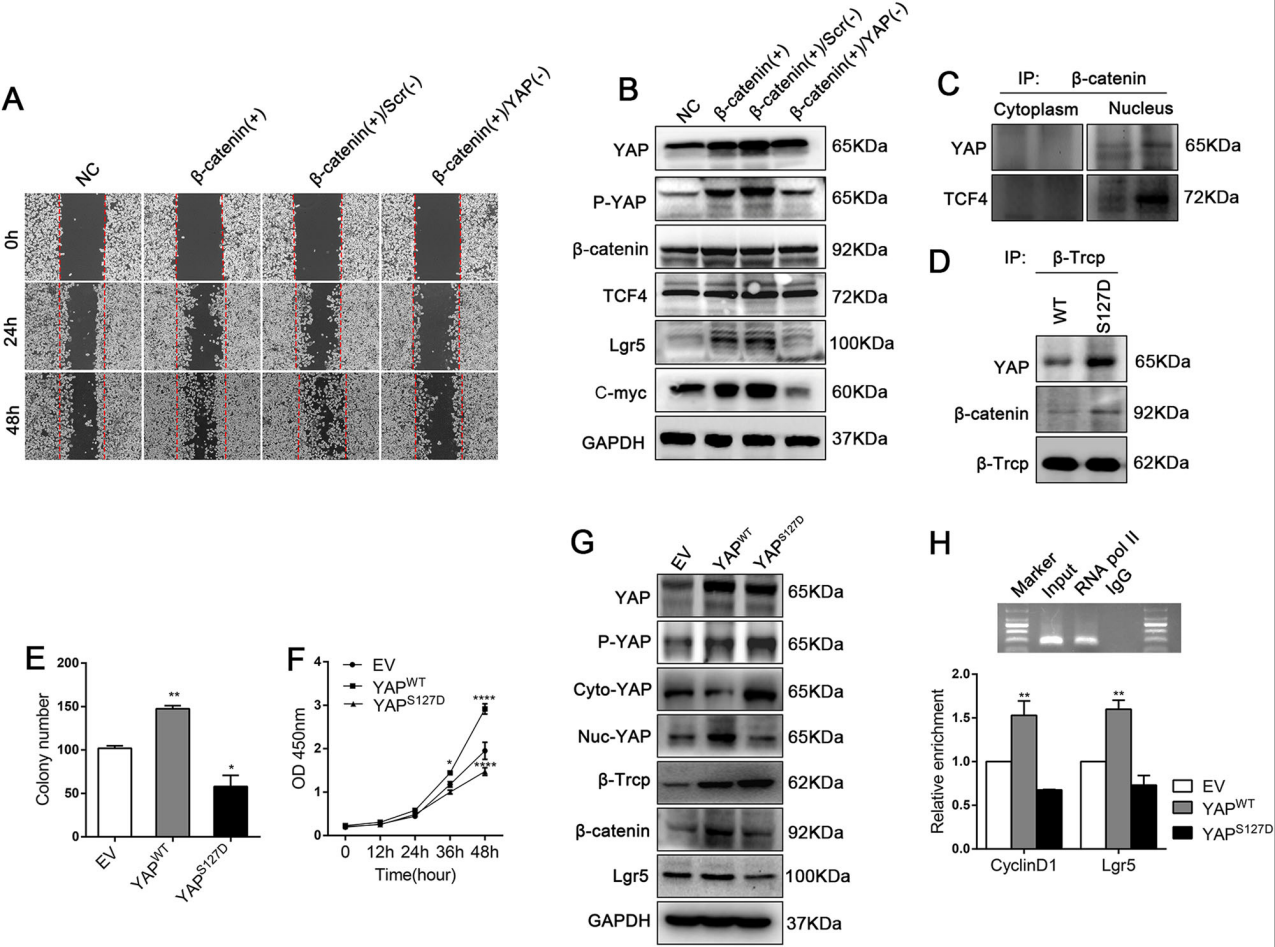
Nuclear YAP and β-catenin/TCF4 formed a transcriptional complex; Lgr5 and cyclin D1 were targets of this complex in epithelial cells. (**a**) Wound-healing detection in FHC cells transfected with negative control (NC) plasmids, β-catenin-overexpressing (β-catenin( + )) plasmids or β-catenin-overexpressing plasmids either plus Scr-siRNA (β-catenin( + )/Scr( – )) or YAP-siRNA (β-catenin( + )/YAP( – )). (**b**) IB analysis of total YAP, P^S127^-YAP and Wnt/β-catenin signalling associated proteins from the above FHC cells after the different treatments. (**c**) IB analysis of endogenous YAP and TCF4 expression in co-IP assay with anti-β-catenin antibody in nuclear and cytosol lysates of FHC cells. (**d**) IB analysis of exogenous YAP and β-catenin expression in co-IP assays with anti-β-Trcp antibody in stable 293T-YAP^WT^ and 293T-YAP^S127D^ cells. (**e**) Cell Counting Kit-8 assay in stable DLD1-EV, DLD1-YAP^WT^ and DLD1-YAP^S127D^ cells. (**f**) Colony formation assay in stable DLD1-EV, DLD1-YAP^WT^ and DLD1-YAP^S127D^ cells from three independent experiments. *P < 0.05, **P < 0.01, ****P < 0.0001. (**g**) IB analysis of total YAP, P^S127^-YAP and Wnt/β-catenin signalling associated proteins in stable DLD1-EV, DLD1-YAP^WT^and DLD1-YAP^S127D^ cells. (**h**) Real-time PCR analysis of cyclin D1 and Lgr5 chromatin-IP with anti-TCF4 antibody from stable FHC-EV, FHC-YAP^WT^ and FHC -YAP^S127D^ cells. Data represent the means ± SEMs. **P < 0.01

By using western blotting, we screened expression of YAP protein in several cell lines, to determine which cell line should be applied to the subsequent lentivirus plasmids (YAP-Low) or siRNAs (YAP-High) transfection (Supplementary Figure 3A). We then investigated the interaction between YAP and β-catenin in FHC clones stably overexpressing YAP (YAP^WT^ mainly expressed in the nucleus, Figs. 5e and 6g) or EV control. Compared with that of cells infected with an EV, YAP^WT^ increased wound healing and significantly suppressed apoptosis after DSS treatment in FHC cells (Fig. 5d,g). The expression of Wnt-associated proteins including β-catenin, Lgr5 and cyclin D1 was upregulated in YAP^WT^ cells compared with that in EV cells (Fig. 5e). Moreover, silencing YAP by siRNA caused a decrease in the expression of Lgr5 in HT29 cells (Fig. 5f). However, silencing β-catenin impeded monolayer wound healing and reversed the up-regulated Wnt transcription in YAP^WT^ FHC cells (Fig. 5d,e). These data indicated that nuclear YAP triggered Wnt/β-catenin signalling to drive IEC proliferation.

### Nuclear YAP and β-catenin/TCF4 formed a transcriptional complex; Lgr5 and cyclin D1 were targets of this complex in epithelial cells

As stated above, YAP effectively promoted the transcriptional activation of Wnt/β-catenin signalling molecules, whereas increased β-catenin was loss of function when YAP was deficient upon inflammation. This prompted us to investigate whether YAP is a component of the β-catenin-regulated transcriptional complex. To test this hypothesis, we first transfected FHC β-catenin-overexpressing cells with YAP-specific siRNA. The result showed that β-catenin overexpression enhanced ‘wound’ closure, but silencing YAP in FHC-β-catenin ( + ) cells attenuated this effect (Fig. 6a). Furthermore, protein expression levels of Wnt targets such as Lgr5 and C-myc were increased in FHC-β-catenin ( + ) cells, but this was completely reversed in FHC-β-catenin ( + )/YAP( – ) cells (Fig. 6b). Moreover, endogenous co-immunoprecipitation (IP) analysis indicated that YAP directly interacted with β-catenin in the cell nucleus, but not in the cytoplasm (Fig. 6c). Chromatin IP detection showed that Lgr5 and cyclin D1 expression was clearly increased in FHC-YAP^WT^ cells (Fig. 6h). Thus, we speculated that YAP is a dispensable part of the β-catenin/TCF4 complex that drives IEC proliferation; Lgr5 and cyclin D1 were confirmed as the targets of this complex.

To further investigate the function of nuclear YAP in colon cells, we then constructed another stable clone in which YAP was phospho-mimetically mutated (YAP^S127D^) in DLD1 cells. Notably, in YAP^S127D^ (serine to aspartic acid at residue 127), YAP is constitutively phosphorylated, leading to its functional cytoplasmic localization (Fig. 6g) and inability to promote transcriptional activation. In our study, we found that both YAP and β-catenin can directly bind to the E3 ubiquitin ligase β-Trcp, and YAP^S127D^ recruited more β-catenin to β-Trcp, causing more β-catenin degradation than in stable 293T-YAP^WT^ cells (Fig. 6d). YAP^S127D^ significantly depressed DLD1 clone formation and cell proliferation (Fig. 6e,f and Supplementary Figure 3B). Accordingly, immunoblotting analysis verified the substantial suppression of β-catenin and Lgr5 protein expression in YAP^S127D^ cells compared that in YAP^WT^ or EV cells (Fig. 6g). In addition, the Wnt transcriptional targets Lgr5 and cyclin D1 were clearly down-regulated in YAP^S127D^ cells (Fig. 6h). Thus, deficiency of nuclear YAP accumulation inactivated Wnt/β-catenin signalling, cytoplasmic phosphor-YAP even mediated suppressive effect. Collectively, nuclear YAP participated in β-catenin/TCF4 transcriptional complex to drive epithelial cell proliferation.

### YAP^WT^ and phospho-mimetic YAP controlled the development of colitis-associated carcinoma in opposite manners

Finally, we performed a classic CAC procedure in mice that were infected with YAP^S112D^ (mouse S112D corresponding to S127D in humans) and YAP^WT^ lentivirus to further probe the effect of YAP on chronic inflammation. After the first cycle, the colon length was 12 mm shorter in the YAP^S112D^ mice than in the YAP^WT^-infected mice (Supplementary Figure 4D). In addition, after chemically induced injury, YAP^S112D^ mice presented with worse intestinal barrier dysfunction than the YAP^WT^ control mice (Supplementary Figure 4E). Moreover, YAP^S112D^ mice exhibited a decrease in Wnt-related protein expression levels (Supplementary Figure 4A and F), combined with more severe destruction including the disappearance of crypt cells and inflammatory cell infiltration in the mucosa (Supplementary Figure 4B and C). Accordingly, compared with YAP^WT^ mice, mutant YAP (YAP^S112D^) expression in the colonic crypts led to an increased susceptibility to colitis induced by short-term AOM/DSS exposure.

**Fig. 7:**
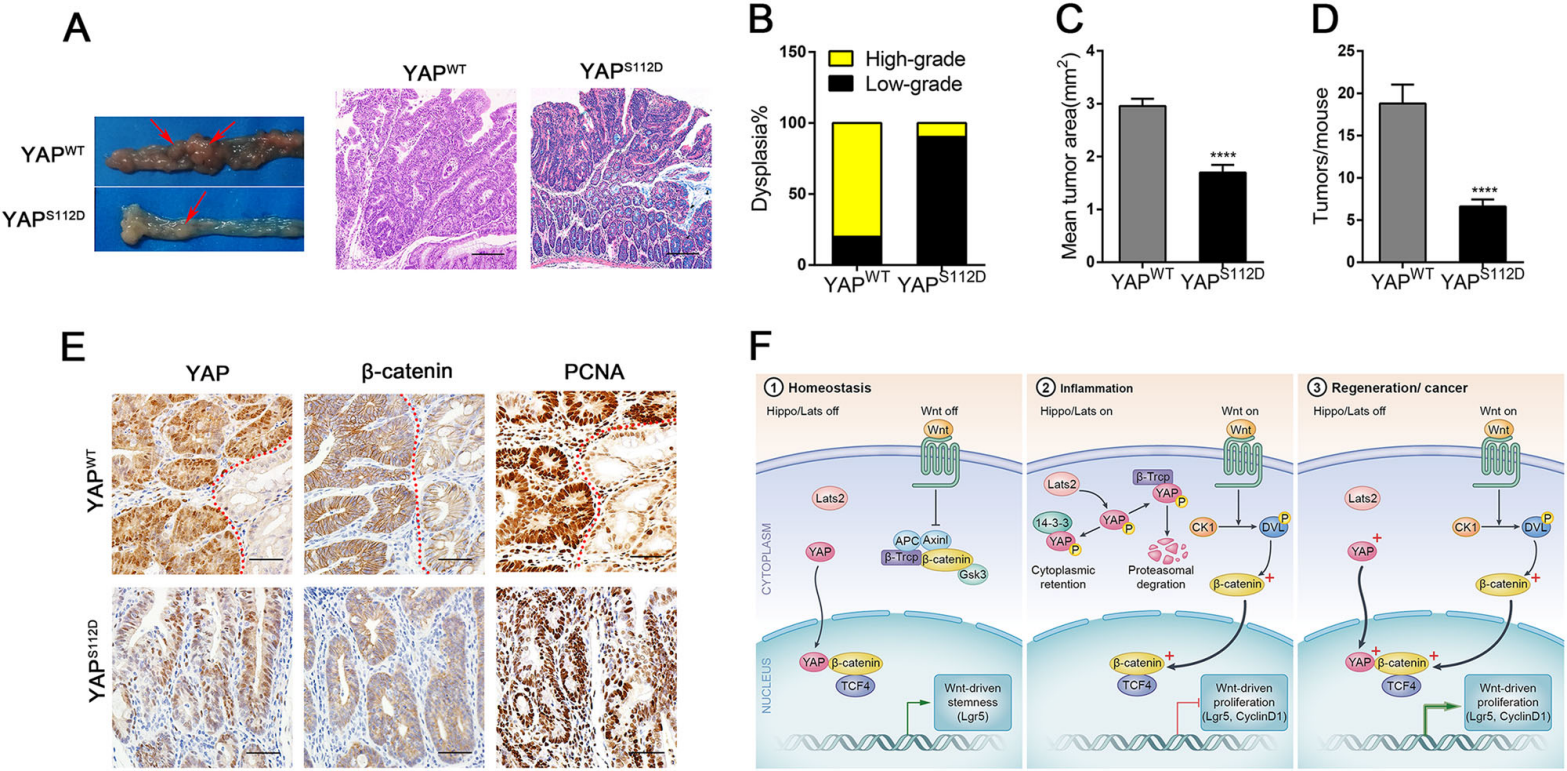
YAP^WT^ and phospho-mimetic YAP regulated the development of colitis-associated carcinoma in opposite manners. YAP^WT^ and mutant YAP^S112D^ mice were obtained by classical CAC procedures and sacrificed at day 75 (*n* = 10 each). (**a**) Left: macroscopic tumours (arrow); right: colon mucosal histology (× 100); (**b**) percentage of mice with dysplasia; (**c**) mean tumour area; (**d**) number of tumours per mouse in YAP^WT^ and YAP^S112D^ mice at day 75. (**e**) Immunohistochemical staining (400 × ) in YAP^WT^ and YAP^S112D^ mice after 75 days of AOM/DSS administration. Red dashed lines represent the boundary between the normal mucosa and neoplastic regions. Data represent the means ± SEMs. Scale bars = 200 μm. ****P < 0.0001. (**f**) Model of how Hippo/YAP and the Wnt/β-catenin signalling pathway work during epithelial homeostasis, inflammation, regeneration and cancer

After the third cycle ended, we had successfully created a CAC model (Fig. 7a) and found a concomitant up-regulation in the nuclear staining of YAP and β-catenin in the neoplastic area of tissues (Fig. 7e). Interestingly, mice infected with YAP^S112D^ developed significantly smaller tumour areas and tumour numbers than YAP^WT^ mice (Fig. 7c,d). In addition, histological assessments showed that the tumours in YAP^WT^ mice were usually identified as having high-grade dysplasia, but the colonic mucosa of YAP^S112D^ mice presented with low-grade dysplasia (Fig. 7b). Therefore, we concluded that YAP promoted the development of associated neoplastic lesions during chronic mucosal injury and regeneration; conversely, phospho-YAP hindered IEC repair, thereby suppressing the excessive growth and development of CAC.

## Discussion

YAP generally acts as a transcriptional co-activator of the Hippo pathway, which is essential for controlling organ size, tissue growth and tumour development.^11,12^ Normally, YAP is located at the crypt base near the ISCs, but its biological function in the intestine has not been clearly documented. UC is a subcategory of IBD and a prominent pathophysiological feature of this disease is impaired epithelial regeneration.^7^ In this study, we found that YAP was specifically downregulated in the colonic crypts of human UC and that mice overexpressing YAP increased IEC proliferation to maintain homeostasis and promote mucosal regeneration. However, YAP also increased colitis-associated carcinoma susceptibility, which is similar to the effect from STAT3 activation^35,36^. We concluded that YAP is a critical regulator of IEC self-renewal, crypt regeneration after inflammation and CAC growth.

Recent studies have investigated YAP as a core component of the Hippo pathway, and its interaction with TEAD family proteins triggers the transcription of target genes, such as CTGF and Areg, to promote proliferation and migration in multiple tumour types^37–40^. Based on the function of YAP in cancer, preceding studies have reported that YAP deficiency impaired DSS-induced intestinal regeneration, but the mechanism of this impairment was not determined^41^. Meanwhile, intestinal gp130 signalling facilitated epithelial cell proliferation and conferred resistance against mucosal invasion through the activation of YAP and Notch^4^. In our investigation, using the DSS-induced colitis and repair model, we found that YAP expression was markedly enhanced after the removal of DSS and was accompanied by actively proliferating colonic epithelial cells in crypts, which revealed that YAP has a crucial role in crypt cell regeneration. This YAP-driven effect on cell proliferation was also verified in YAP-overexpressing mice that YAP^WT^ mice exhibited a greater IEC self-renewal ability and thus resistance to colitis damage than EV-infected mice. More importantly, YAP substantially promoted crypt regeneration after inflammatory stimulation.

Owing to the similar roles of YAP and Wnt in cell proliferation, we studied the relationship between YAP and Wnt/β-catenin signalling. Wnt is essential in stem cell maintenance, cell survival and proliferation^19,42^. The β-catenin/TCF4 complex^40,43^ has a central role in modulating the expression of its target genes. As reported, the deletion of Lgr5^+^ cells after radiation-induced damage and the subsequent regeneration period resulted in extensive crypt loss and the disappearance of villus structure^44^. In our study, during the regeneration following inflammation, we were amazed to find that the nuclear co-localization of YAP and β-catenin was increased. Overexpressing YAP significantly improved IEC ‘wound-healing’ ability and increased the expression of both β-catenin and the transcriptional targets of Wnt signalling, whereas silencing β-catenin attenuated these effects, indicating that YAP promoted IEC proliferation through the activation of Wnt/β-catenin signalling.

Next, to probe the regulation between YAP and β-catenin, the deeper mechanism of these effects was investigated. Previous literature has demonstrated that YAP/TAZ inhibited the CK1 δ/ε-mediated phosphorylation of DVL in the cytoplasm, thereby suppressing the activation of Wnt/β-catenin^45^. In addition, YAP/TAZ-TEAD induced the secretion of Wnt inhibitors such as Wnt 5A/B and suppressed Wnt/β-catenin activation^46^. However, nuclear YAP may also interact with β-catenin on Sox2 and Snai2 genes to promote cardiomyocyte proliferation and influence heart size^47^. In our research, we found the YAP could directly interact with β-catenin in the nucleus to form the YAP/β-catenin/TCF4 transcriptional complex in colon cells, moreover, Lgr5 and cyclin D1 were confirmed as the targets of this complex. To further clarify the function of nuclear YAP in IEC proliferation, we established a phospho-mimetic mutant of YAP (resulting in YAP retention in the cytoplasm) and found that it markedly inhibited Wnt activation by recruiting more β-Trcp to the β-catenin destruction complex. Meanwhile, cancer cell proliferation and tumour development were suppressed in the phosphor-YAP mutant. Therefore, nuclear YAP participated in the β-catenin/TCF4 transcriptional complex in colon cells and the activity of this complex was normal during homeostasis but was dramatically enhanced during colitis-induced regeneration and carcinoma (Fig. 7f).

In summary, insufficient regenerative proliferation increases the risk for disease including IBD and age-related tissue atrophy, while persistent and excessive proliferation may cause oncogenesis. Early mucosal healing is particularly important in the treatment of IBD. In this study, YAP-driven IEC proliferation regulated epithelial regeneration after inflammation, which might be caused by adaptive modulation of the nuclear YAP/β-catenin/TCF4 complex. Collectively, YAP has potential to serve as a preventative therapeutic tool for the treatment of UC. Conversely, the systemic delivery of mutant YAP to target tumours in Wnt-addicted cancers provides new selectivity for colon cancer therapy.

## Electronic supplementary material

Supplementary files
