# Supplementary material for: YAP triggers the Wnt/β-catenin signalling pathway and promotes enterocyte self-renewal, regeneration and tumorigenesis after DSS-induced injury
Source: Cell Death Dis. 2018 Feb 2;9(2):153. doi: 10.1038/s41419-017-0244-8 (PMC5833613; doi:10.1038/s41419-017-0244-8)
Supplement: Supplementary file 1 — Supplementary files [file 41419_2017_244_MOESM1_ESM.docx]

**Supplementary figures and figure legends**


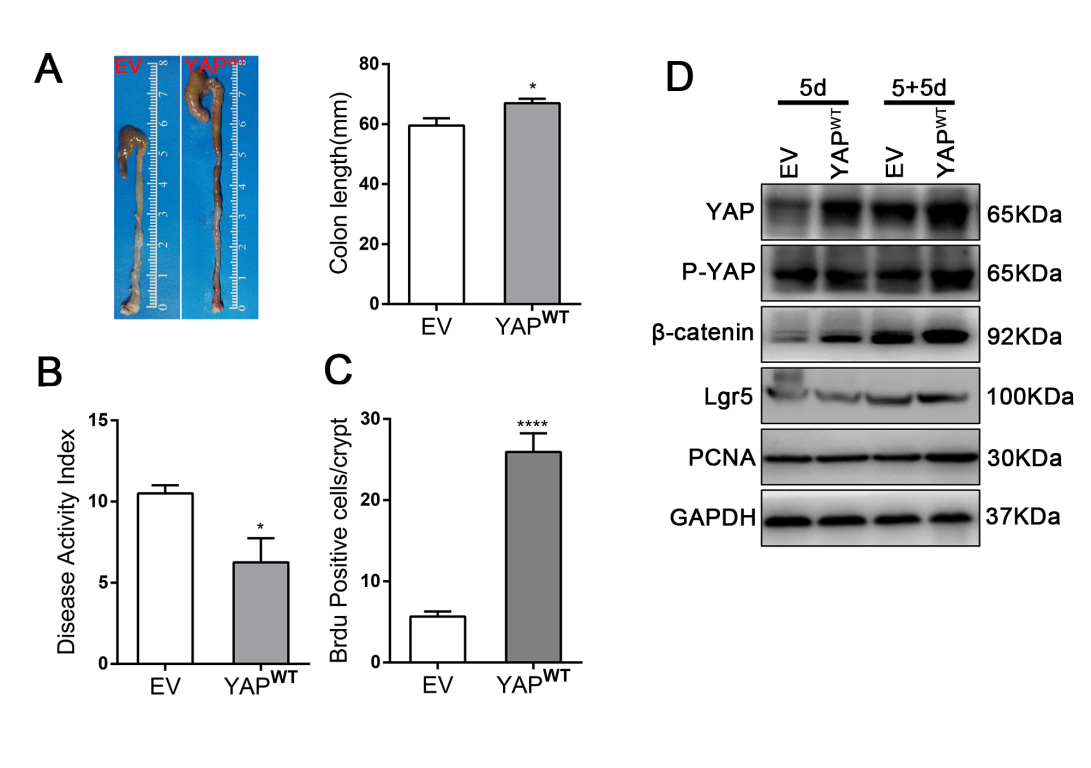


Supplementary figure 1. YAP over-expression played protective role during DSS colitis and promoted mucosal healing. (A) Colon length and (B) Disease activity index of YAP^WT^ and EV mice after a 5-day DSS treatment. (C) Brdu positive cells were enumerated per crypt of 5+5d YAP^WT^ and EV mice. Data are mean ± SEM. *P<0.05, ****P<0.0001. (D) IB analysis of colonic lysates prepared from isolated enterocytes of 5d and 5+5d YAP^WT^ and EV mice for the indicated proteins’ expression.


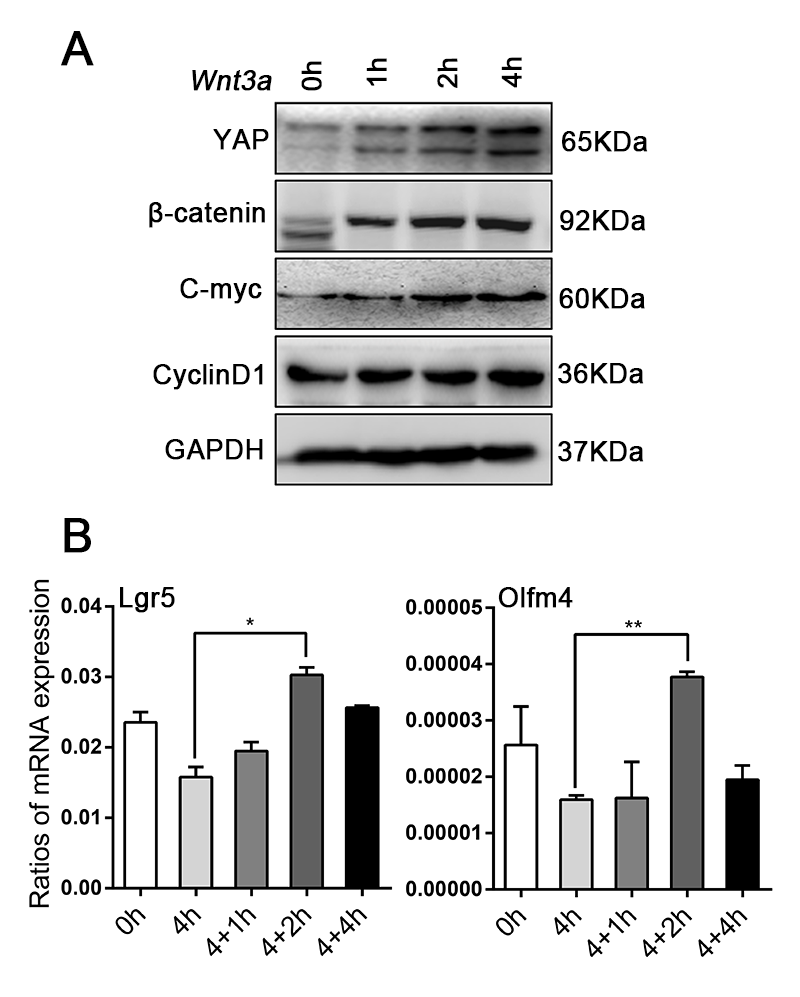


Supplementary figure 2. YAP increased IECs regeneration after inflammation *in vitro*. (A) IB analysis for the indicated proteins in FHC cells. Cells were serum-starved overnight and then stimulated with *Wnt 3a* (100 ng/ml) at the indicated time. (B) mRNA levels of *Lgr5* and *Olfm4* in inflammation and repair model of FHC cell lines. Data are mean ± SEM. *P<0.05, **P<0.01.


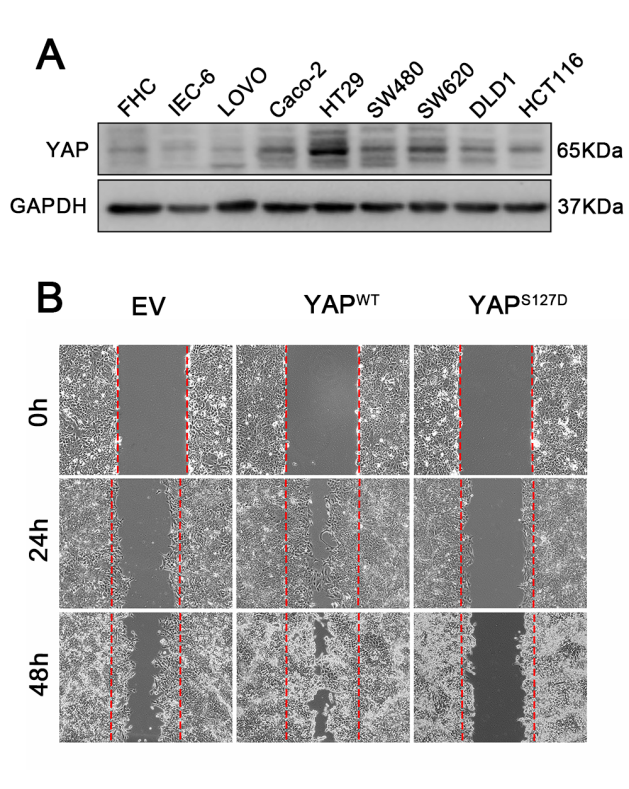


Supplementary figure 3. YAP expression in intestinal cell lines and YAP^WT^ facilitated colonic cells proliferation. (A) Western blot analysis of total YAP protein expression level in intestinal cell lines. (B) Wound healing detection of DLD1-EV, DLD1-YAP^WT^and DLD1-YAP^S127D^ cells at 0h, 24h and 48h.


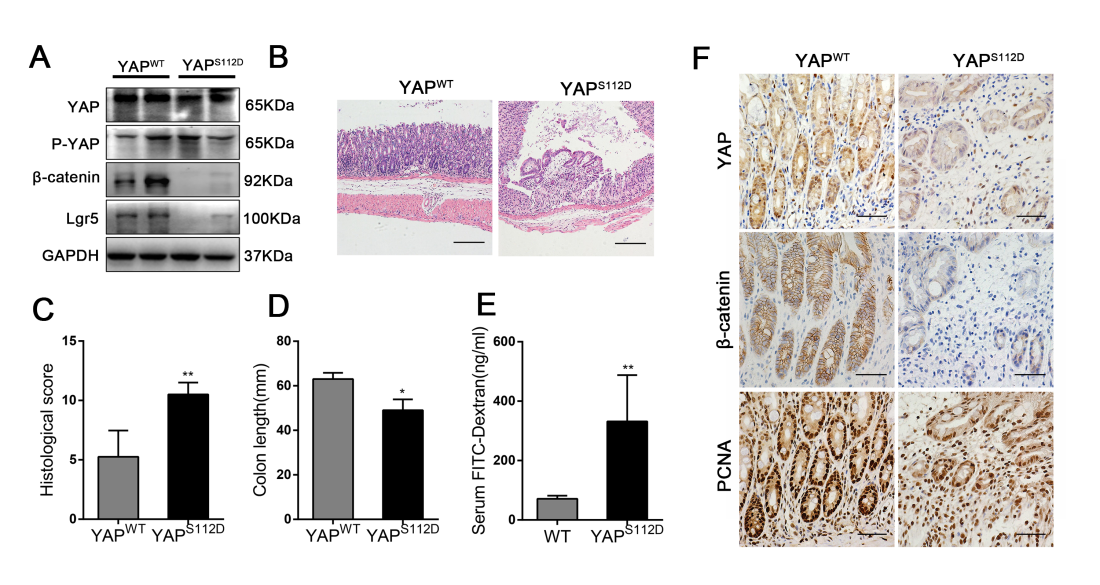


Supplementary figure 4. YAP^WT^ and phospho-mimetic YAP controlled development of colitis associated carcinoma in an opposite manner. YAP^WT^ and mutant YAP^S112D^ mice were obtained classical CAC administration and sacrificed at day 14(n=4 each). (A) IB analysis for the indicated antibodies in primary colonic cells prepared from YAP^WT^ and YAP^S112D^ mice at day 14. (B) Colon mucosal histology (100 X); (C) Histological scores; (D) Colon length; (E) Serum FITC-Dextran of YAP^WT^ and YAP^S112D^ mice. (F) Immunohistochemical staining (400X) for YAP, [β-catenin](http://www.baidu.com/link?url=AhpQmu0JvAUPSU68B0UGo-tMrqYtvqyOe57BQvbQdAXGvzX8kwQ7YsDyK_MMRjN4wwyAp3PhpZrmnrqyb-j4d3U6d9B2pjOEvpe2hx8z_IG) and PCNA in YAP^WT^ and YAP^S112D^ mice at day 14 of AOM/DSS administration. Data are mean ± SEM. Scale bars = 200μm. *P<0.05, **P<0.01,

**Supplementary table1.** Specific primer sequences of Q-PCR(5’→3’)

| hYAP-F’ | CTCGGCTTCAGGTCCTCTTC |
| --- | --- |
| hYAP-R’ | AGGGTCAAGCCTTGGGTCTA |
| hLgr5-F’ | CAACCTCAGCGTCTTCACCT |
| hLgr5-R’ | CATCCAGACGCAGGGATTGA |
| hAscl2-F’ | CTCGACCTATGAGCCTCAGC |
| hAscl2-R’ | CACTGCAGTCGAGAAGCTGT |
| hOlfm4-F’ | AGCTGGAGGTGGAGATAAGAA |
| hOlfm4-R’ | CCACAGACGGTTTGCTGATG |
| mYAP-F’ | ATTTCGGCAGGCAATACGGA |
| mYAP-R’ | CGCTGTCTGTGCTCTCATCT |
| mIL-6-F’ | CTCTGCAAGAGACTTCCATCCA |
| mIL-6-R’ | GACAGGTCTGTTGGGAGTGG |
| mLgr5-F’ | AGCGTCTTCACCTCCTACCT |
| mLgr5-R’ | ATCTAGGCGCAGGGATTGAAG |

**Supplementary table2.** Specific sequences of siRNAs(5’→3’)

| hYAP-sense | GGUCAGAGAUACUUCUUAATT |
| --- | --- |
| hYAP-antisense | UUAAGAAGUAUCUCUGACCTT |
| hCTNNB1-sense | GCUCAUCAUACUGGCUAGUTT |
| hCTNNB1-antisense | ACUAGCCAGUAUGAUGAGCTT |
| Negative control-sense | UUCUCCGAACGUGUCACGUTT |
| Negative control -antisense | ACGUGACACGUUCGGAGAATT |
